# Telomere maintenance in African trypanosomes

**DOI:** 10.3389/fmolb.2023.1302557

**Published:** 2023-11-24

**Authors:** Bibo Li

**Affiliations:** ^1^ Center for Gene Regulation in Health and Disease, Department of Biological, Geological, and Environmental Sciences, College of Arts and Sciences, Cleveland State University, Cleveland, OH, United States; ^2^ Case Comprehensive Cancer Center, Case Western Reserve University, Cleveland, OH, United States; ^3^ Department of Inflammation and Immunity, Lerner Research Institute, Cleveland Clinic, Cleveland, OH, United States; ^4^ Center for RNA Science and Therapeutics, Case Western Reserve University, Cleveland, OH, United States

**Keywords:** telomere, telomere end processing, PolIE, telomerase, *Trypanosoma brucei*, antigenic variation, telomeric and subtelomeric DNA recombination

## Abstract

Telomere maintenance is essential for genome integrity and chromosome stability in eukaryotic cells harboring linear chromosomes, as telomere forms a specialized structure to mask the natural chromosome ends from DNA damage repair machineries and to prevent nucleolytic degradation of the telomeric DNA. In *Trypanosoma brucei* and several other microbial pathogens, virulence genes involved in antigenic variation, a key pathogenesis mechanism essential for host immune evasion and long-term infections, are located at subtelomeres, and expression and switching of these major surface antigens are regulated by telomere proteins and the telomere structure. Therefore, understanding telomere maintenance mechanisms and how these pathogens achieve a balance between stability and plasticity at telomere/subtelomere will help develop better means to eradicate human diseases caused by these pathogens. Telomere replication faces several challenges, and the “end replication problem” is a key obstacle that can cause progressive telomere shortening in proliferating cells. To overcome this challenge, most eukaryotes use telomerase to extend the G-rich telomere strand. In addition, a number of telomere proteins use sophisticated mechanisms to coordinate the telomerase-mediated *de novo* telomere G-strand synthesis and the telomere C-strand fill-in, which has been extensively studied in mammalian cells. However, we recently discovered that trypanosomes lack many telomere proteins identified in its mammalian host that are critical for telomere end processing. Rather, *T. brucei* uses a unique DNA polymerase, PolIE that belongs to the DNA polymerase A family (*E. coli* DNA PolI family), to coordinate the telomere G- and C-strand syntheses. In this review, I will first briefly summarize current understanding of telomere end processing in mammals. Subsequently, I will describe PolIE-mediated coordination of telomere G- and C-strand synthesis in *T. brucei* and implication of this recent discovery.

## Introduction

Eukaryotic genomes typically contain linear chromosomes, at the ends of which lie the telomere nucleoprotein complexes (Lu et al., 2013; Srinivas et al., 2020). In most eukaryotes, telomeres consist of simple repetitive sequences with the G-rich strand running 5′ to 3′ towards the chromosome end (Cross et al., 1989; Riethman et al., 1989; Podlevsky et al., 2008; Lyčka et al., 2023). Telomeres are essential for genome integrity and chromosome stability (O'Sullivan and Karlseder, 2010; Meena et al., 2015; Borges et al., 2022; Hackett et al., 2001; Murnane, 2010), mainly because telomeres form a specialized structure, masking the natural chromosome ends from DNA damage repair machineries and preventing the telomeric DNA from nucleolytic degradation and illegitimate DNA processes such as DNA recombination (de Lange, 2005; Muraki et al., 2012; Stewart et al., 2012; Ruis and Boulton, 2021). Proper chromosome end protection requires telomere proteins that directly or indirectly bind to the telomeric DNA (de Lange, 2005; Giraud-Panis et al., 2010b). Critically short telomeres cannot serve as an adequate docking site for telomere proteins, resulting in an “unprotected” telomere, which is recognized by the DNA damage response machinery to arrest cell growth. Therefore, most eukaryotic cells cannot continue to multiply (they enter replicative senescence) when telomeres are critically short (Shay and Wright, 2005; Aubert and Lansdorp, 2008; Vaiserman and Krasnienkov, 2020), and telomere maintenance mechanisms are essential for continued cell proliferation (Greider, 1998; Lulkiewicz et al., 2020; Rossiello et al., 2022). Conventional DNA polymerases always require a template and a primer and synthesize DNA in the 5′ to 3′ polarity, so they cannot fully replicate the ends of linear DNA molecules, leading to progressive telomere shortening with each round of DNA replication (Wellinger, 2014; Bonnell et al., 2021). Most eukaryotes use telomerase, a specialized reverse transcriptase to synthesize the telomere G-rich strand DNA *de novo*, thereby solving this “end replication” problem (Greider and Blackburn, 1987; Lingner et al., 1995; Shay and Wright, 2019). The telomerase core enzyme has a protein subunit, TERT, that has the reverse transcriptase catalytic activity, and an RNA subunit, TR, that provides the template for telomeric DNA synthesis (Autexier and Lue, 2006; Wyatt et al., 2010). Many telomere proteins control telomere length by regulating the access of telomerase to its telomere substrate (Smogorzewska and de Lange, 2004; Jafri et al., 2016; Aramburu et al., 2020).

In multicellular organisms such as humans, maintaining genome stability is critical for organism fitness as it helps suppress tumorigenesis (Guo et al., 2023). However, several eukaryotic pathogens that undergo antigenic variation would welcome a certain degree of telomere/subtelomere plasticity (Li, 2012). *Trypanosoma brucei* that causes human African trypanosomiasis, *Plasmodium falciparum* that causes malaria, and *Pneumocystis jirovecii* that causes pneumonia in immunodeficient patients are common in that they undergo antigenic variation to evade the host’s immune response (Li, 2012; Li, 2021), and genes encoding their major surface antigens involved in antigenic variation are located at subtelomeric regions (Stringer and Keely, 2001; Ralph et al., 2005; Schmid-Siegert et al., 2017; Cosentino et al., 2021). Similarly, in opportunistic pathogen *Candida glabrata* that causes mucosal and systemic infections in immunodeficient patients, the *EPA* gene family is also located at subtelomeric regions, where *EPA* encodes surface glycoproteins called epithelial adhesins that are important for host-pathogen interaction (De Las Penas et al., 2003).

Telomere dysfunction can induce genome instability (Hackett et al., 2001; Murnane, 2010). In addition, telomeres and subtelomeres behave like fragile sites and frequently experience increased levels of replication fork stalling and recombination (Sfeir et al., 2009; Glover et al., 2013; Zhang et al., 2013; Glousker and Lingner, 2021; Mirceta et al., 2022). Hence, locating the major surface antigen genes or contingency genes at subtelomeres presumably facilitate antigenic variation and/or adaptation of the microbial pathogen to its environment (Barry et al., 2003; López-Fuentes et al., 2018; Rahnama et al., 2021; Xu et al., 2021; Contreras et al., 2022). Specifically in *T. brucei*, variant surface glycoprotein (VSG) is the major surface antigen when the parasite proliferates in its mammalian host in extracellular spaces (Cross, 1975; Mugnier et al., 2016; Silva Pereira et al., 2022). *T. brucei* sequentially expresses distinct VSGs on its surface to evade the host’s immune response (Horn, 2014; McCulloch et al., 2017). While all >2,500 *VSG* genes and pseudogenes are located at subtelomeric regions (many in long *VSG* gene arrays) (Berriman et al., 2005; Cross et al., 2014; Müller et al., 2018), only those in VSG expression sites (ESs), polycistronic transcription units immediately upstream of the telomere repeats (de Lange and Borst, 1982; Berriman et al., 2002; Hertz-Fowler et al., 2008), are transcribed by RNA polymerase I in a strictly monoallelic manner (Cross, 1975; Gunzl et al., 2003; Gunzl et al., 2015). VSG switching can occur at the transcription level where the originally active *VSG* ES is silenced while a new one is derepressed (Myler P. et al., 1984; Myler P. J. et al., 1984). VSG switching can also occur through DNA recombination, where a new *VSG* sequence replaces the original active *VSG* sequence (Myler P. et al., 1984; Myler P. J. et al., 1984; Rudenko et al., 1996; McCulloch et al., 1997; Rudenko et al., 1998; Robinson et al., 1999). DNA double strand breaks (DSBs) at the active *VSG* vicinity has been shown to be a potent trigger for VSG switching (Alsford et al., 2009; Boothroyd et al., 2009; Glover et al., 2013; Li, 2021), as homology directed DNA recombination (HDR) repairs DSBs most accurately (Jasin and Rothstein, 2013; Haber, 2018; Wright et al., 2018), and HDR is very active in *T. brucei* (McCulloch and Barry, 1999; Robinson et al., 1999; Conway et al., 2002a; Barnes and McCulloch, 2007; Glover et al., 2008; Marin et al., 2018). Consistently, for known essential *T. brucei* telomere proteins, a transient depletion of the protein leads to an increased amount of DNA breaks at the telomere and subtelomere and more frequent VSG switching, indicating that these telomere proteins help maintain the telomere stability and suppress VSG switching (Jehi et al., 2014a; Jehi et al., 2014b; Jehi et al., 2016; Nanavaty et al., 2017; Afrin et al., 2020a; Afrin et al., 2020b; Saha et al., 2021; Rabbani et al., 2022; Gaurav et al., 2023). Furthermore, in telomerase null cells where the active ES-adjacent telomere is extremely short, a significant increase in the VSG switching rate is observed (Hovel-Miner et al., 2012). In *P. falciparum*, many *var* genes that encode the major surface antigen *Pf*EMP1 involved in antigenic variation are also located at subtelomeres (Rubio et al., 1996; Hernandez-Rivas et al., 1997). In addition, DNA recombination frequently occurs at *P. falciparum* subtelomeres and contributes to divergence of *var* gene families (Calhoun et al., 2017), and this subtelomere plasticity is enhanced when a DSB is introduced (Calhoun et al., 2017; Zhang et al., 2019b). Nevertheless, although subtelomere plasticity facilitates antigenic variation, telomere maintenance in these eukaryotic parasites is also essential for genome integrity and parasite survival (Li et al., 2005; Yang et al., 2009; Glover et al., 2013; Jehi et al., 2014b; Jehi et al., 2016; Nanavaty et al., 2017; Afrin et al., 2020b; Saha et al., 2021; Rabbani et al., 2022; Gaurav et al., 2023). For example, inducing a DSB near or within the active *VSG* is detrimental to *T. brucei*, as only <10% of the population survives through VSG switching (Glover et al., 2013). Therefore, telomere/subtelomere plasticity is a double-edged sword, and telomere maintenance is essential for cell proliferation of eukaryotic parasites (Li, 2021). Interestingly, telomere maintenance mechanisms are not identical in the protozoan parasite *T. brucei* and its mammalian host.

### A brief overview of telomere maintenance in mammalian cells

**FIGURE 1:**
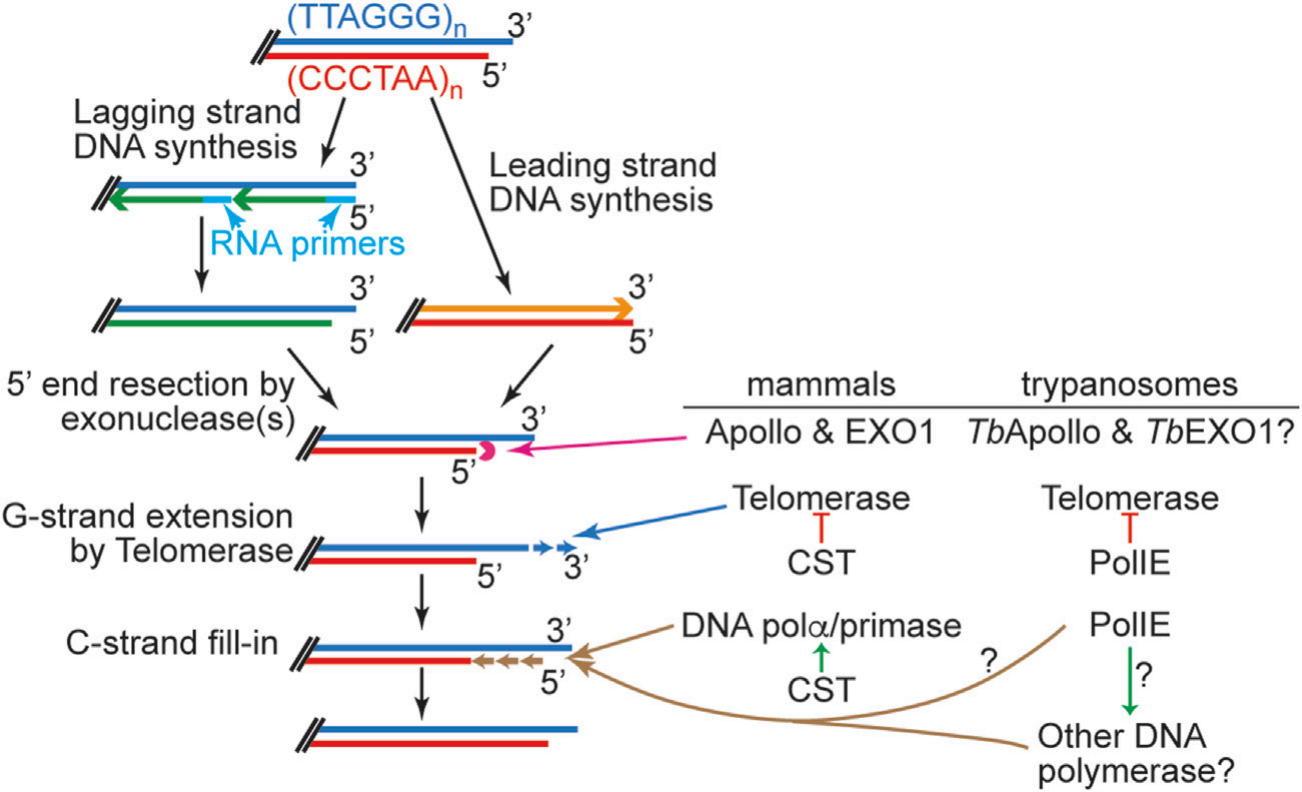
Left, A simplified summary of various steps in telomere end processing. Right, mammalian and trypanosome key telomere proteins involved in each step are listed. Enzymes directly involved in DNA degradation/elongation/replication are linked to the corresponding process with a long arrow. Red lines with a bar at the end stand for inhibitory effects. Short green arrows stand for stimulatory effects. Speculated functions are indicated with question marks.

Telomere replication has multiple steps (Wu et al., 2012; Maestroni et al., 2017; Bonnell et al., 2021). The chromosome internal portion of the telomere can be replicated by conventional DNA polymerases during the S phase with the help from DNA helicases such as BLM, WRN, and RTEL1 (Opresko et al., 2002; Crabbe et al., 2004; Flanary, 2004; Lillard-Wetherell et al., 2004; Machwe et al., 2004; Uringa et al., 2011; Uringa et al., 2012; Vannier et al., 2013; Zimmermann et al., 2014) that disrupt the G-quadruplex structure formed by the telomere G-rich sequence (Gilson and Géli, 2007; Higa et al., 2017; Bryan, 2020). However, telomere ends are processed in a special way (Figure 1) (Lundblad, 2012; Bonetti et al., 2014). Mammalian telomere end processing has been extensively studied, although some regulatory mechanisms are not fully understood. After DNA replication by conventional DNA polymerases, the leading strand DNA replication product has a blunt end, while the lagging strand DNA replication results in a short 3′ overhang after the last primer is removed (Figure 1). Therefore, with each round of DNA replication by conventional DNA polymerase, telomere shortens progressively, hence the “End Replication Problem”. While telomerase can synthesize the G-rich telomere DNA *de novo*, it poses another problem. Telomerase uses single-stranded DNA with G-rich sequence as its substrate (Wallweber et al., 2003; Schmidt and Cech, 2015), so the blunt telomere end from the leading strand DNA replication cannot directly serve as a substrate for telomerase. In mammalian cells, these blunt ends are first converted by Apollo, a 5′ to 3′ exonuclease, to an end with a short 3′ overhang (van Overbeek and de Lange, 2006; Wu et al., 2010; Wu et al., 2012). Subsequently, Exo1, another 5′ to 3′ exonuclease, resects more 5′ telomere DNA, generating a longer 3′ overhang structure at both telomeres of the same chromosome (Wu et al., 2012). This allows telomerase to bind to the telomere end and extend the G-rich strand by *de novo* DNA synthesis. Subsequently, the C-rich telomere strand can be extended by DNA Polα/primase using the G-rich strand as template in a process called “C-strand fill-in” (Bonnell et al., 2021; Olson et al., 2022; He and Lim, 2023).

**FIGURE 2:**
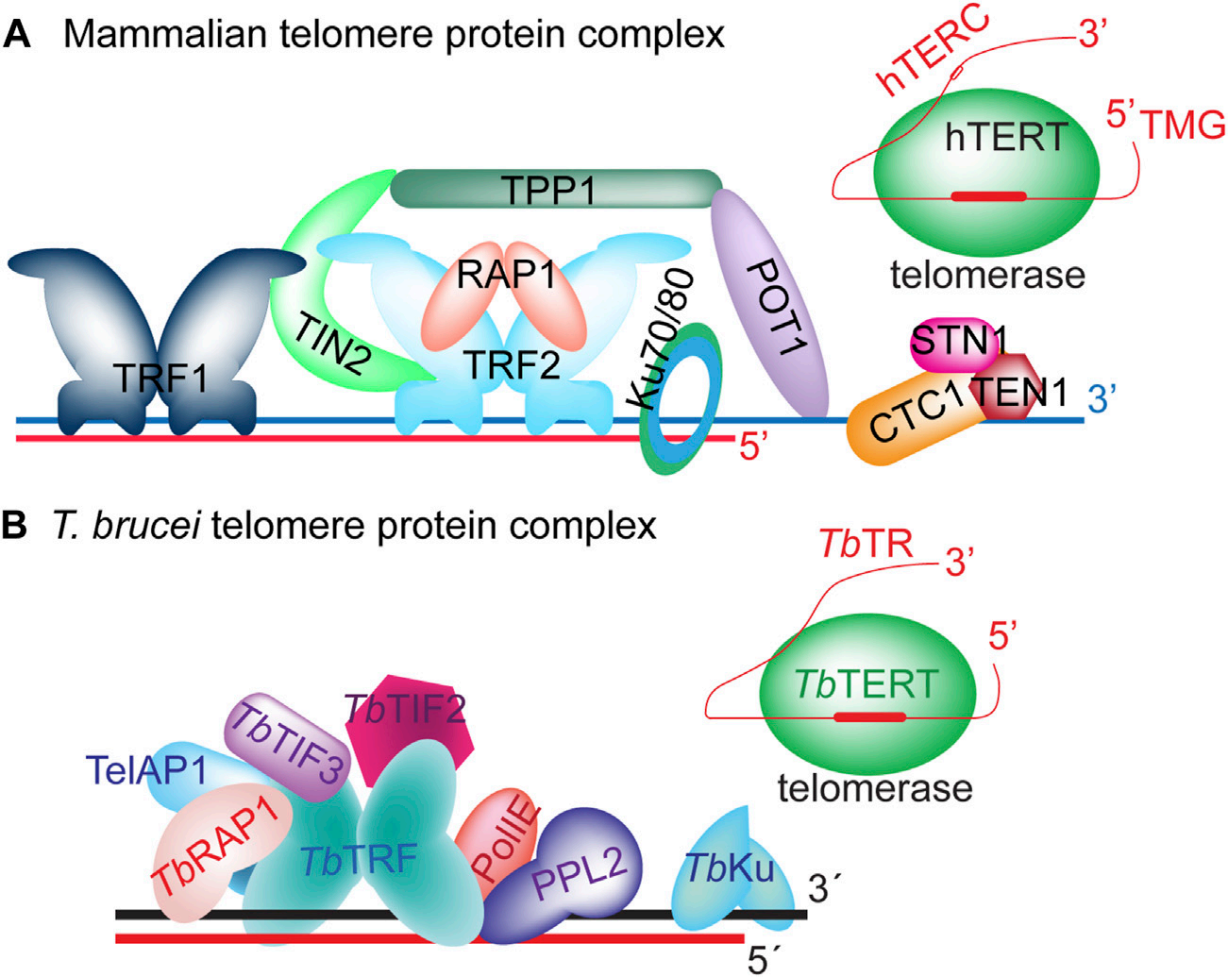
The telomere protein complex in mammals **(A)** and in *T. brucei* **(B)**.

Telomere length is heavily regulated by telomere proteins (Kim et al., 1999; Smogorzewska et al., 2000; Li and de Lange, 2003; Hockemeyer et al., 2005; Mason and Skordalakes, 2010; Takai et al., 2016; Aramburu et al., 2020; Bonetti et al., 2020). In mammalian cells, the Shelterin and CST complexes are key proteins associated with the telomere and play essential roles in telomere maintenance (Figure 2) (Liu et al., 2004a; de Lange, 2005; Giraud-Panis et al., 2010a; Lim and Cech, 2021). Shelterin has six protein components (de Lange, 2005): TRF1 (Chong et al., 1995) and TRF2 (Bilaud et al., 1997; Broccoli et al., 1997) bind to the duplex telomeric DNA (Bianchi et al., 1997; Bilaud et al., 1997; Broccoli et al., 1997), RAP1 (Li et al., 2000) interacts with TRF2, TPP1 (Liu et al., 2004b; Ye et al., 2004b; Houghtaling et al., 2004) and POT1 (Baumann and Cech, 2001; Baumann et al., 2002; Loayza and de Lange, 2003) bind the single-stranded telomere 3′ overhang as a heterodimer (Hwang et al., 2012; Xu et al., 2019), while TIN2 (Kim et al., 1999) interacts with TRF1, TRF2, and TPP1 (Ye et al., 2004a; O'Connor et al., 2006; Houghtaling et al., 2004) and serves as a bridge linking various Shelterin components together. The CST complex has three protein components: CTC1 (or CDC13 in budding yeast), STN1, and TEN1 (Rice and Skordalakes, 2016; Cai and de Lange, 2023). They bind single-stranded telomeric DNA as a trimer (Bhattacharjee et al., 2017; Cai et al., 2022), which structurally resembles the RPA complex (Miyake et al., 2009; Rice and Skordalakes, 2016; Barbour and Wuttke, 2023). Interestingly, TPP1, POT1, and CST components all have OB folds (Xin et al., 2008; Zhong et al., 2012; Bhattacharjee et al., 2016; Rice et al., 2017; Wang et al., 2023), which are commonly used to bind ssDNA or RNA (Theobald et al., 2003; Horvath, 2011; Nguyen et al., 2020).

Among OB fold-containing telomere proteins, TPP1 is a key telomerase recruiting factor (Rajavel et al., 2014; Sexton et al., 2014). The TEL patch domain of TPP1 directly interacts with TERT and helps recruit telomerase to the telomere end (Nandakumar et al., 2012; Grill et al., 2019; Sekne et al., 2022). TPP1 also helps stimulate telomerase processivity (Latrick and Cech, 2010; Nandakumar et al., 2012; Sandhu et al., 2021). On the other hand, POT1 seems to be able to both stimulate and inhibit telomerase action (Kelleher et al., 2005; Xu et al., 2019; Aramburu et al., 2020; Gu et al., 2021; Zade and Khattar, 2023). Binding of CST to the telomere 3′ overhang also inhibits the access of telomerase to its telomere substrate, hence suppressing telomere G-strand synthesis (Chen et al., 2012; Chen et al., 2013; Chen and Lingner, 2013; Feng et al., 2018; Zaug et al., 2021). On the other hand, CST promotes the telomere C-strand fill-in process by recruiting DNA Polα/primase to the telomere and specifying the origins for telomeric C-strand replication (Zaug et al., 2022).

### Unique features of telomere maintenance in African trypanosomes

Protozoan parasites such as *T. brucei* and *P. falciparum* are eukaryotic pathogens and have linear genomes, but their telomere protein complexes are not completely conserved as those found in their mammalian host. In *P. falciparum*, SIR2 (Freitas-Junior et al., 2005; Merrick and Duraisingh, 2007; Mancio-Silva et al., 2008; Tonkin et al., 2009; Religa and Waters, 2012) and HP1 (Perez-Toledo et al., 2009; Hernandez-Rivas et al., 2010) homologs have been identified to associate with the telomere chromatin and help suppress subtelomeric gene expression. SIR2 homologs are histone deacetylases that help establish/maintain the heterochromatin structure (Xu et al., 2007; Jing and Lin, 2015), and HP1 homologs associate with heterochromatin and promote heterochromatin formation/maintenance/propagation (Zeng et al., 2010; Schoelz and Riddle, 2022). In addition, *Pf*TRZ has been identified to bind the duplex telomeric DNA, subtelomeric *var* genes, and 5S rDNA loci (Bertschi et al., 2017). *Pf*TRZ is also a remote functional homologue of TFIIIA and regulates 5S rRNA expression (Bertschi et al., 2017). *Pf*AP2Tel is another protein identified to bind the telomeric DNA (Sierra-Miranda et al., 2017), and it contains an atypical AP2 domain that is frequently found in transcription factors of Apicomplexans (Painter et al., 2011). Finally, *Pf*GBP2 has been identified to bind the telomere G-quadruplex structure and G-rich RNAs (Edwards-Smallbone et al., 2022). However, only *Pf*TRZ has been shown to have an essential function in telomere maintenance (Bertschi et al., 2017). Functions of *Pf*AP2Tel and *Pf*GBP2 at the telomere are unclear. Nothing is known about telomere end processing in *P. falciparum* except that telomerase is a major factor for telomere maintenance (Figueiredo et al., 2005).

Much more has been learned about the *T. brucei* telomere protein complex and its function in telomere maintenance (Li, 2010; Li, 2015; Li, 2021; Li and Zhao, 2021). As shown in Figure 1, a number of proteins are involved in telomere end processing at various steps in mammalian cells. Similar processes are expected in *T. brucei*. Specifically, *T. brucei* has Apollo and EXO1 homologs, although their roles in telomere end processing have not been investigated. In addition, both *T. brucei* telomerase components, *Tb*TERT and *Tb*TR, have been identified, and it has been shown that telomerase-mediated telomere synthesis is the major mechanism of telomere maintenance (Dreesen et al., 2005; Gupta et al., 2013; Sandhu et al., 2013). Interestingly, *T. brucei* telomere length grows continuously during cell proliferation (Bernards et al., 1983), which is different from that in mammalian cells, where telomere lengths are maintained within a limited size range (Teixeira et al., 2004; Hug and Lingner, 2006). Therefore, *T. brucei* telomerase action is likely regulated by different mechanisms than that in mammals. Furthermore, the *T. brucei* Ku70/80 complex is also essential for proper telomere lengthening but not for telemeric silencing (Conway et al., 2002b; Janzen et al., 2004). Interestingly, although Ku homologs are typically involved in non-homologous end-joining pathway (NHEJ) (Frit et al., 2019), *T. brucei* only has HDR and microhomology-mediated end-joining (MMEJ) but not NHEJ (Burton et al., 2007; Glover et al., 2011). On the other hand, yeast and human Ku homologs have been shown to interact with the telomerase RNA directly and contribute to telomerase recruitment to the telomere (Stellwagen et al., 2003; Ting et al., 2005; Pfingsten et al., 2012; Hass and Zappulla, 2015). It is possible that *T. brucei* Ku has a similar function.

The *T. brucei* Shelterin-equivalent protein complex includes a TRF homolog (*Tb*TRF) (Li et al., 2005), a RAP1 homolog (*Tb*RAP1) (Yang et al., 2009), and *Tb*TIF2 that is a functional homolog of mammalian TIN2 (Jehi et al., 2014b) (Figure 2). *Tb*TRF directly interacts with *Tb*RAP1 (Yang et al., 2009), *Tb*TIF2 (Jehi et al., 2014b), and likely PolIE, too (Rabbani et al., 2022). Whether *Tb*TRF recruits RecQ family DNA helicases (such as BLM and WRN) to the telomere to facilitate telomeric repeat replication as its mammalian homologs (Opresko et al., 2002; Crabbe et al., 2004; Lillard-Wetherell et al., 2004; Machwe et al., 2004; Zimmermann et al., 2014) is unclear. *Tb*RECQ2, a RecQ helicase, has been shown to help repair DNA damage, and loss of *Tb*RECQ2 leads to an increase in VSG switching rate (Devlin et al., 2016). Among *T. brucei* Shelterin components, *Tb*RAP1 is a key regulator of *VSG* monoallelic expression, as depletion of *Tb*RAP1 results in global *VSG* derepression for up to 1,000-fold, and *Tb*RAP1’s dsDNA binding activity is essential for *VSG* silencing (Yang et al., 2009; Pandya et al., 2013; Afrin et al., 2020a; Afrin et al., 2020b). Since *Tb*RAP1 helps compact telomere and subtelomere chromatin (Pandya et al., 2013), it is hypothesized that *Tb*RAP1-mediated telomeric silencing is an epigenetic effect that relies on chromatin structure remodeling, although the underlying mechanism is not fully clear. Unexpectedly, *Tb*RAP1 binds the active *VSG* RNA through its RRM domain (not present in vertebrate and yeast RAP1 homologs), which is essential for the full-level expression of the active *VSG* (Gaurav et al., 2023). *Tb*TRF and *Tb*RAP1 also help maintain the telomere/subtelomere stability by suppressing levels of telomere repeat-containing RNA (TERRA) (Rudenko and Van der Ploeg, 1989) and telomeric R-loop (Nanavaty et al., 2017; Saha et al., 2021). TERRA is expressed from the active *VSG* ES-adjacent telomere (Nanavaty et al., 2017; Saha et al., 2021), and it can form the three-stranded R-loop structure with the duplex telomeric DNA (Fernandes et al., 2021), while R-loops are known to have a tendency of inducing DNA breaks (Mackay et al., 2020; Brickner et al., 2022). Indeed, overexpressing RNaseH1 that degrades the RNA in the RNA:DNA hybrid in *Tb*RAP1/*Tb*TRF-depleted cells can reduce the level of telomeric R-loops, the amount of telomeric DNA break, and the VSG switching rate (Nanavaty et al., 2017; Saha et al., 2021). Therefore, it is clear that excessive amount of TERRA and telomeric R-loops reduces the telomere/subtelomere stability by causing more DNA damage and increases the VSG switching rate, at least transiently (Nanavaty et al., 2017; Saha et al., 2021). In contrast, it is unknown whether depletion of TERRA has any detrimental effect on parasite proliferation or telomere functions, as depletion of TERRA is difficult to achieve. Nevertheless, *Tb*TRF and *Tb*RAP1 probably do not use identical mechanisms to regulate TERRA and telomeric R-loop levels, as *Tb*TRF can directly bind TERRA through its C-terminal Myb domain (that also binds the duplex telomeric DNA) while *Tb*RAP1 does not bind TERRA (Li et al., 2005; Saha et al., 2021; Gaurav et al., 2023). *Tb*TIF2 has also been shown to help maintain telomere/subtelomere stability by suppressing DNA breaks at the telomere vicinity (Jehi et al., 2014b), and *Tb*TIF2 and *Tb*TRF have both overlapping and distinct roles (Jehi et al., 2016), although *Tb*TIF2’s effect on TERRA has not been examined in detail.

Additional telomere proteins have also been identified in *T. brucei* that do not seem to have any homologs in mammals (Figure 2). TelAP1 is identified through its ability to bind a telomere sequence-containing DNA fragment and as a component in the *Tb*TRF protein complex (Reis et al., 2018). It is not essential for *T. brucei* proliferation but plays a role in *T. brucei* differentiation from the infectious form proliferating in its mammalian host to the insect form proliferating in the midgut of its insect vector (Reis et al., 2018). So far, no telomere-specific OB fold-containing ssDNA binding factors have been identified in *T. brucei* (Rabbani et al., 2022). The *T. brucei* telomere chromatin has been purified and analyzed by Proteomics of Isolated Chromatin segments (PICh) (Dejardin and Kingston, 2009; Rabbani et al., 2022). In addition, the *Tb*TRF-*Tb*TIF2 protein complex has been purified and examined (Rabbani et al., 2022). Furthermore, proteins that directly bind the telomeric repeats were identified using a telomere sequence-containing DNA fragment (Reis et al., 2018). These studies helped identify *T. brucei* telomere proteins comprehensively, but no obvious homologs of TPP1, POT1, and CST have been identified, although further structural analysis may eventually identify homologs of these OB fold-containing telomere proteins. On the other hand, *Leishmania* RPA1 can bind the telomere ssDNA (Neto et al., 2007; Da Silveira et al., 2013; Pavani et al., 2014; Fernandes et al., 2020), and *Tb*RPA1 is part of the telomere chromatin as shown by PICh (Rabbani et al., 2022), suggesting that *Tb*RPA may fulfill at least part of CST functions, particularly since the CST trimeric complex structurally resembles the RPA complex (Miyake et al., 2009; Rice and Skordalakes, 2016; Barbour and Wuttke, 2023). However, more investigation is necessary to reveal *Tb*RPA’s function in telomere end processing, if any.

Interestingly, two DNA polymerases have been identified to associate with the *T. brucei* telomere chromatin (Reis et al., 2018; Rabbani et al., 2022). One of these, PolIE, has been shown to coordinate telomere G- and C-strand syntheses, fulfilling similar functions as mammalian CST and DNA Polα/primase (Rabbani et al., 2022). PolIE is an A-type DNA polymerase that has been identified in the *Tb*TRF protein complex and in the telomere chromatin (Rabbani et al., 2022). It also binds the telomere sequence-containing DNA fragment (Reis et al., 2018). Initially, PolIE was annotated to be a DNA polymerase theta homolog. However, careful sequence analysis indicates that PolIE actually belongs to the DNA polymerase I family (type A family) (Leal et al., 2020). PolIE is essential for *T. brucei* survival and participates in DNA damage repair (Leal et al., 2020; Rabbani et al., 2022). However, sequence alignment indicates that PolIE lacks the domains in human Polθ that are associated with lesion bypass activity (Leal et al., 2020), suggesting that PolIE lacks a translesion DNA synthesis activity (Leal et al., 2020), although this has not been experimentally confirmed.

PolIE can be depleted efficiently (>90%) by RNAi within 24 h, and PolIE-depleted cells quickly stop proliferating, indicating that PolIE is essential for *T. brucei* cell growth (Leal et al., 2020; Rabbani et al., 2022). However, PolIE-depleted cells do not arrest at specific stage of the cell cycle (Leal et al., 2020; Rabbani et al., 2022). Nevertheless, PolIE helps to repair DNA damage caused by UV irradiation, MMS, and cisplatin treatment, as PolIE-depleted cells survive more poorly than WT cells when treated with these DNA damaging agents (Leal et al., 2020; Rabbani et al., 2022). Depletion of PolIE also leads to a mild VSG derepression and an increased VSG switching rate, with increased amount of DNA damage at the telomere and subtelomere (Leal et al., 2020; Rabbani et al., 2022). Further analysis indicated that PolIE is essential for telomere end processing (Rabbani et al., 2022).

**FIGURE 3:**
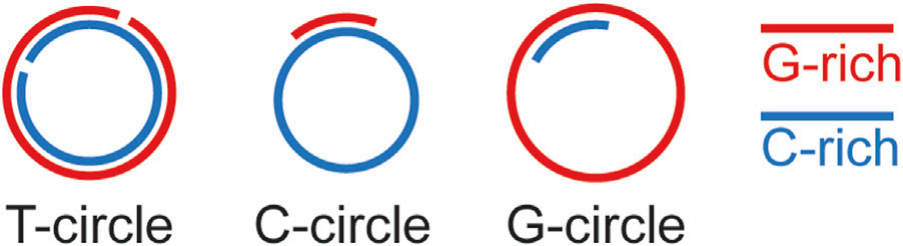
Diagrams of telomere circles (T-circles) and telomeric C and G-circles.

*T. brucei* telomeres have the expected terminal telomere 3′ overhang structure, but this overhang appears to be very short (∼12 nt in asynchronized cells) (Sandhu and Li, 2011), suggesting that G-rich and C-rich telomeric DNA syntheses are well coordinated with each other. Theoretically, the length of the telomere 3′ overhang changes at different steps of telomere end processing (Figure 1), and longer telomere 3′ overhang has been detected in S phase in yeast (Dionne and Wellinger, 1996; Fridholm et al., 2013). However, changes in telomere 3′ overhang length throughout the cell cycle have not been reported in *T. brucei* as synchronizing *T. brucei* cells under a physiological condition is challenging. The telomerase activity is also required for the telomere 3′ overhang structure (Sandhu and Li, 2017), indicating that telomerase is a major contributing factor for elongating the telomere 3′ overhang. The first sign suggesting that PolIE plays an important role in telomere end processing came from the observation that PolIE-depleted cells have a dramatically elongated telomere 3′ overhang, indicating an abnormal coordination between telomere G- and C-strand syntheses (Rabbani et al., 2022). It is known that telomeres in *T. brucei* can form a T-loop structure (Munoz-Jordan et al., 2001), although it is unknown whether T-loops can form at any stage of the cell cycle or only during a limited window in the cell cycle. Apparently, T-loop formation depends on the telomere 3′ overhang, and longer overhang is more prone to invade the duplex telomeric DNA. In addition, resolution of the T-loop can lead to formation of telomere circles (T-circles, Figure 3) (Pickett et al., 2009). Indeed, depletion of PolIE results in an increased amount of T-circles and telomere C-circles (Rabbani et al., 2022), where T circles are mostly double stranded with nicks on both strands, and C-circles are mostly single stranded (Figure 3) (Pickett et al., 2009; Henson et al., 2017). It is worth to mention that telomere C-circles are typically detected in ALT cells (Henson et al., 2009; Zhang et al., 2019a; Chen et al., 2021), which are telomerase negative human cancer cells that use alternative mechanisms to maintain their telomere length (Zhang and Zou, 2020). A high level of telomere C-circles typically suggests frequent telomere recombination, which has also been shown to be a key mechanism for telomere maintenance in ALT cells (Zhang and Zou, 2020). In PolIE-depleted cells, longer telomere 3′ overhang (Rabbani et al., 2022) presumably has a higher probability to invade the duplex telomere DNA region and leads to an increased T-loop formation, while resolution of the T-loop structure will result in circular DNA species. Depending on the exact invasion site and subsequent migration of the crossover region, this could lead to recombination events involving both telomeric and subtelomeric sequences. The increased amount of T-circles and C-circles in PolIE-depleted cells and the fact that PolIE depletion causes an increased VSG switching rate all suggest that PolIE normally helps suppress DNA recombination at the telomere and subtelomere by limiting the length of telomere 3′ overhang (Rabbani et al., 2022).

Using EdU-labeling to examine newly synthesized telomere G- and C-strand DNA levels, Rabbani et al. further discovered that the level of telomere G-strand synthesis is increased upon depletion of PolIE (Rabbani et al., 2022). Furthermore, this increase is telomerase-dependent (Rabbani et al., 2022). Therefore, PolIE normally suppresses telomerase action of elongating the telomere G-rich strand DNA. Interestingly, PolIE depletion-induced increase in the telomere 3′ overhang length and the telomere C-circle level is not telomerase-dependent, indicating that PolIE has additional functions other than suppressing telomerase (Rabbani et al., 2022). Telomere C-strand fill-in also affects the telomere 3′ overhang length, and depletion of PolIE causes a subtle decrease in nascent telomere C-strand DNA, suggesting that PolIE can also stimulate telomere C-strand fill-in (Rabbani et al., 2022).

*T. brucei* PolIE negatively affects telomere G-strand elongation and stimulates telomere C-strand fill-in (Rabbani et al., 2022), suggesting that it behaves similarly as mammalian CST. However, PolIE does not seem to be equivalent to CST. The main functions of CST in telomere end processing are two-fold. First, binding of CST on the telomere 3′ overhang physically sequesters the telomere terminal ssDNA and prevents telomerase from accessing the telomere substrate, hence inhibiting telomerase-mediated telomere G-strand synthesis (Zaug et al., 2021). PolIE also suppresses the telomerase action (Rabbani et al., 2022), but the underlying mechanisms are unclear. A recent proteomic study did not detect any interaction between *Tb*TERT and PolIE (Davis et al., 2023), suggesting that PolIE may not directly affect the telomerase activity. On the other hand, PolIE, as a DNA polymerase, is expected to be able to bind the telomere DNA, as it is required for a proper telomere C-strand fill-in (Rabbani et al., 2022). However, whether PolIE also binds the telomere 3′ overhang and masks this substrate from telomerase binding is unknown.

Second, CST recruits DNA Polα/primase to the telomere and specify origins of DNA replication to complete the telomere C-strand fill-in (Zaug et al., 2022; He and Lim, 2023), but CST itself does not have DNA polymerase activity (Zaug et al., 2022). Sequence analysis showed that PolIE has the conserved DNA polymerase domain (Leal et al., 2020), suggesting that it does have a DNA polymerase activity, although this has not been experimentally verified. On the other hand, PolIE only has limited homology to the domains of human Polθ that are associated with the lesion bypass activity (Leal et al., 2020), suggesting that PolIE is not a translesion DNA polymerase, although further investigation is necessary to validate this. Therefore, PolIE may be directly involved in telomere C-strand synthesis, the same as human Polα/primase. However, whether the DNA polymerase activity of PolIE is required for telomere C-strand fill-in is unclear. It is also possible, though unlikely, that PolIE simply acts as CST and recruits another DNA polymerase to the telomere to actually fulfill the C-strand fill-in function.

Interestingly, *T. brucei* Polymerase-Primase Like 2 (PPL2) has also been identified from all three proteomic approaches described above aimed to identify telomere proteins (Reis et al., 2018; Rabbani et al., 2022), strongly suggesting that PPL2 is yet another telomere protein in *T. brucei*. PPL2 is essential and has translesion DNA polymerase activities that catalyzes error-prone bypass of 6-4 photoproduct, but it lacks a primase activity (Rudd et al., 2013). In addition, PPL2 is important for finishing DNA replication in the G2 phase (Rudd et al., 2013). These observations suggest that PPL2 may also be involved in telomere C-strand fill-in, which occurs after bulk DNA replication in S phase. Further investigation on PPL2’s function in telomere end processing will help us better understand mechanisms of telomere maintenance in this important eukaryotic pathogen.

In summary, telomere end processing (Figure 1) appears to be conserved from *T. brucei* to its mammalian host. However, *T. brucei* telomere protein complex has several distinct components than the human telomere complex. Even though *Tb*TRF, *Tb*RAP1, and *Tb*TIF2 have telomere functions that are more comparable to their mammalian homologs, the sequence homology between these proteins and their mammalian counterparts is only limited within a few functional domains (Li et al., 2005; Yang et al., 2009; Jehi et al., 2014b). In addition, recently identified PolIE and PPL2 do not seem to have any strict homologs in the mammalian telomere complex. Nevertheless, *T. brucei* telomere proteins play essential roles in telomere maintenance, and further investigation will help us better understand how different eukaryotes use different players to achieve the same goals.

## Discussion

Kinetoplastids are ancient organisms diverged from the mammalian branch in the evolutionary tree more than 500 million years ago. Recent studies on *T. brucei* telomere maintenance mechanisms suggest that TRF, RAP1, and TIN2 homologs have been evolved much earlier than TPP1/POT1 and CST homologs (Rabbani et al., 2022). Specifically, the DNA polymerase involved in telomere C-strand fill-in in *T. brucei* is a type A DNA polymerase that arose and diversified in the kinetoplastids lineage (Leal et al., 2020). Whether its ancestral is more closely related to PolI-like or Polθ/ν -like DNA polymerase is unclear. Nevertheless, a strictly conserved PolIE homolog appears to be absent in mammals.

*T. brucei* undergoes antigenic variation. Hence, vaccination is not effective, and chemotherapy is a key approach for treating African trypanosomiasis. Although subtelomere plasticity facilitates antigenic variation, telomere stability is critical for genome integrity and parasite proliferation. As *T. brucei* clearly uses distinct and essential telomere factors than its mammalian host for telomere end processing, the *T. brucei*-unique telomere proteins are promising targets for anti-trypanosome agents. Better understanding about the degree of effects of telomere plasticity and stability on VSG switching will further help us identify anti-parasite means that have clear detrimental effects on parasite growth without enhancing antigenic variation efficiency inadvertently. In addition, *T. cruzi* and *Leishmania* are kinetoplastid parasites that cause devastating human diseases affecting more than ten million people worldwide. They are closely related to *T. brucei,* and homologs of all essential telomere proteins are present in *T. cruzi* and *Leishmania*. It is expected that telomere maintenance mechanisms among these three trypanosomes are highly conserved, and knowledge gleaned from *T. brucei* on telomere protein functions and telomere end processing would also help eradication of *T. cruzi* and *Leishmania.*
